# Autoreactive Effector/Memory CD4^+^ and CD8^+^ T Cells Infiltrating Grafted and Endogenous Islets in Diabetic NOD Mice Exhibit Similar T Cell Receptor Usage

**DOI:** 10.1371/journal.pone.0052054

**Published:** 2012-12-14

**Authors:** Ramiro Diz, Alaina Garland, Benjamin G. Vincent, Mark C. Johnson, Nicholas Spidale, Bo Wang, Roland Tisch

**Affiliations:** Department of Microbiology and Immunology, University of North Carolina, Chapel Hill, North Carolina, United States of America; La Jolla Institute for Allergy and Immunology, United States of America

## Abstract

Islet transplantation provides a “cure” for type 1 diabetes but is limited in part by recurrent autoimmunity mediated by β cell-specific CD4^+^ and CD8^+^ T cells. Insight into the T cell receptor (TCR) repertoire of effector T cells driving recurrent autoimmunity would aid the development of immunotherapies to prevent islet graft rejection. Accordingly, we used a multi-parameter flow cytometry strategy to assess the TCR variable β (Vβ) chain repertoires of T cell subsets involved in autoimmune-mediated rejection of islet grafts in diabetic NOD mouse recipients. Naïve CD4^+^ and CD8^+^ T cells exhibited a diverse TCR repertoire, which was similar in all tissues examined in NOD recipients including the pancreas and islet grafts. On the other hand, the effector/memory CD8^+^ T cell repertoire in the islet graft was dominated by one to four TCR Vβ chains, and specific TCR Vβ chain usage varied from recipient to recipient. Similarly, islet graft- infiltrating effector/memory CD4^+^ T cells expressed a limited number of prevalent TCR Vβ chains, although generally TCR repertoire diversity was increased compared to effector/memory CD8^+^ T cells. Strikingly, the majority of NOD recipients showed an increase in TCR Vβ12-bearing effector/memory CD4^+^ T cells in the islet graft, most of which were proliferating, indicating clonal expansion. Importantly, TCR Vβ usage by effector/memory CD4^+^ and CD8^+^ T cells infiltrating the islet graft exhibited greater similarity to the repertoire found in the pancreas as opposed to the draining renal lymph node, pancreatic lymph node, or spleen. Together these results demonstrate that effector/memory CD4^+^ and CD8^+^ T cells mediating autoimmune rejection of islet grafts are characterized by restricted TCR Vβ chain usage, and are similar to T cells that drive destruction of the endogenous islets.

## Introduction

Type 1 diabetes (T1D) is characterized by the autoimmune destruction of the insulin-secreting β cells residing in the pancreatic islets of Langerhan’s [1]–[5]. In humans and the NOD mouse, a spontaneous model for T1D, β cell autoimmunity is viewed as a chronic inflammatory response mediated by autoreactive CD4^+^ and CD8^+^ T cells [6]–[10]. Initiation of the diabetogenic response involves T cell recognition of a limited number of β cell autoantigens. As β cell autoimmunity progresses, several autoantigens are targeted due to intra- and inter-molecular epitope spread, resulting in the expansion of multiple clonotypes of pathogenic β cell-specific effector T cells (Teff) [11]–[18]. The latter is evident by a T cell receptor (TCR) repertoire marked by expression of multiple TCR variable (V) genes by islet resident T cells [19]–[21], and β cell-specific T cell clones [19], [22]–[26]. Once ∼80% of the β cell mass has been destroyed and/or rendered nonfunctional, hyperglycemic blood levels are achieved and the onset of overt diabetes diagnosed.

Islet transplantation is one approach to replace β cells and restore euglycemia in T1D patients [27]–[29]. Short-term efficacy has been obtained in chronic T1D patients receiving an islet transplant and immunosuppressive drugs. However, widespread application of islet transplantation is limited by a variety of factors, including the persistence of autoreactive T cells which destroy the grafted β cells [6], [9], [10], [30], [31]. A better understanding of the nature of the pathogenic β cell-specific T cells and the response associated with recurrent autoimmunity is critical for the development of immunotherapies that promote long-term islet graft-specific tolerance. Currently, it is unclear whether the same clonotypes of β cell-specific CD4^+^ and CD8^+^ Teff drive destruction of both endogenous and grafted β cells. Our earlier work analyzing TCR Vα and Vβ gene usage by Major Histocompatibility Complex (MHC) class I tetramer-sorted CD8^+^ T cells specific for islet-specific glucose-6-phosphatase catalytic subunit-related protein derived peptide (IGRP_206–214_) indicated that islet graft destruction was mediated by clonotypes also prevalent in the pancreas of the diabetic NOD recipients [32]. However, whether this is a general observation for all β cell-specific CD8^+^ Teff has yet to be established. Furthermore, the clonotypic composition of β cell-specific CD4^+^ Teff mediating islet graft destruction has not been defined. Due to the several known and potential unknown autoantigens driving T1D, analysis of β cell-specific T cell populations by tetramer analysis is cumbersome and impractical to address these key issues.

Accordingly, we have employed a novel multi-parameter flow cytometry approach to determine the TCR Vβ usage by CD4^+^ and CD8^+^ T cells infiltrating grafted and endogenous islets in individual diabetic NOD mice. The approach is advantageous since TCR Vβ usage can be readily assessed for different subsets of CD4^+^ and CD8^+^ T cells residing in multiple tissues of an individual animal. Herein we show that both CD4^+^ and CD8^+^ effector/memory T cells (Teff/mem) infiltrating an islet graft and the pancreas exhibit restricted TCR Vβ usage. Notably, whereas TCR Vβ usage by islet graft-infiltrating CD8^+^ Teff/mem is highly variable among individual animals, TCR Vβ12 is preferentially expressed by CD4^+^ Teff/mem in the majority of NOD recipients. Importantly, TCR Vβ usage by CD4^+^ and CD8^+^ Teff/mem is most similar between grafted and endogenous islets compared to peripheral lymphoid tissues in an individual NOD recipient. These results suggest that immunodominant β cell-specific T cells attacking the endogenous pancreas also mediate islet graft destruction.

## Results

### Defining TCR Vβ Repertoire Usage by Multi-parameter Flow Cytometry

**Figure 1:**
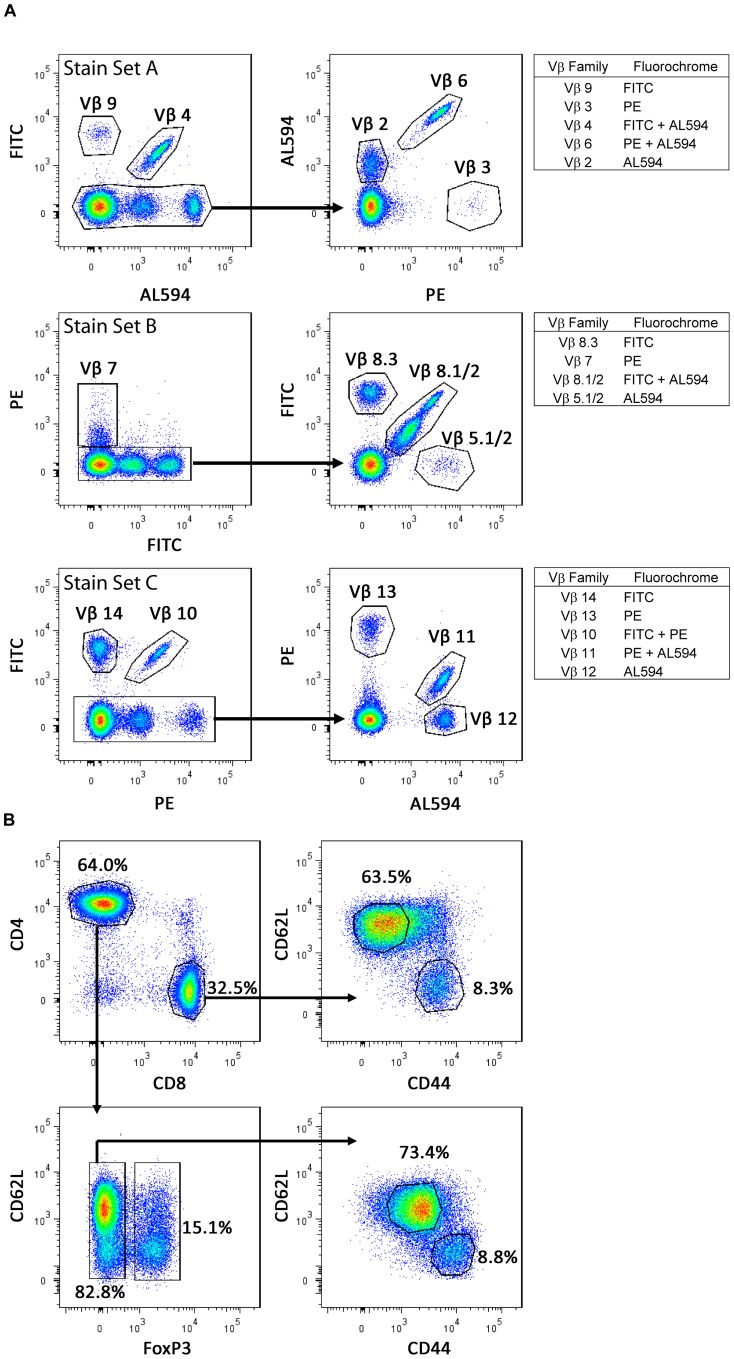
Multiple TCRs can be examined in a pool of T cells. (A) TCR Vβ usage was determined with 3 different stain sets (A, B and C) (see Materials and Methods). Three colors were used – FITC, PE, and streptavidin Alexa594, to visualize up to 6 different TCR Vβ chains per sample. (B) Representative gating scheme in which splenic CD4^+^ and CD8^+^ T cells (CD90.2^+^) were divided into naïve (CD62L^hi^CD44^lo^) and Teff/mem (CD62L^lo^CD44^hi^) subsets. CD4^+^ T cells were further defined based on FoxP3-expression.

A modified multi-parameter flow cytometry method was used to identify single TCR Vβ populations using combinations of fluorochrome- and biotinylated-labeled antibodies specific for TCR Vβ chains in individual diabetic NOD mice receiving syngeneic islet grafts [33]–[36]. Three different staining panels were designed in which up to 6 different TCR Vβ chains could be detected simultaneously for a given staining set (Figure 1A). Typically, a small non-reactive population, defined as T cells expressing TCR Vβ chains for which antibodies are unavailable, was also present. In general ≥75% of all CD4^+^ and CD8^+^ T cells stained with the anti-Vβ antibody panel used (Tables 1,2,3,4).

CD4^+^ and CD8^+^ T cells were co-stained with antibodies specific for CD90.2 (Thy1.2), CD44 and CD62L to identify eff/mem (CD44^hi^ CD62L^lo^) and naïve (CD62L^hi^ CD44^lo^) T cell subsets [37]–[39]. The majority of T cells exhibited one of these two phenotypes (Figure 1B). Although CD44 did not permit distinction between Teff and Tmem, activated and naïve T cells were readily discerned. Foxp3-expressing immunoregulatory T cells (Treg) were also distinguished from naïve and eff/mem CD4^+^ T cells in the analyses by intracellular staining of Foxp3 (Figure 1B).

Diabetic NOD female mice (blood glucose levels >250 mg/dL) were implanted with 500 syngeneic NOD.*scid* islets under the kidney capsule. Ten days post-implantation, a time at which recurrent diabetes is first detected, TCR Vβ usage by CD4^+^ and CD8^+^ naïve and Teff/mem was examined in the islet graft, the renal lymph node (RLN) draining the graft site, pancreas, pancreatic lymph node (PLN), and spleen of individual recipients.

### Naive T cells Infiltrating Grafted and Endogenous Islets Exhibit TCR Vβ Chain Usage Similar to Naïve T cells in Lymphoid Tissues

**Figure 2:**
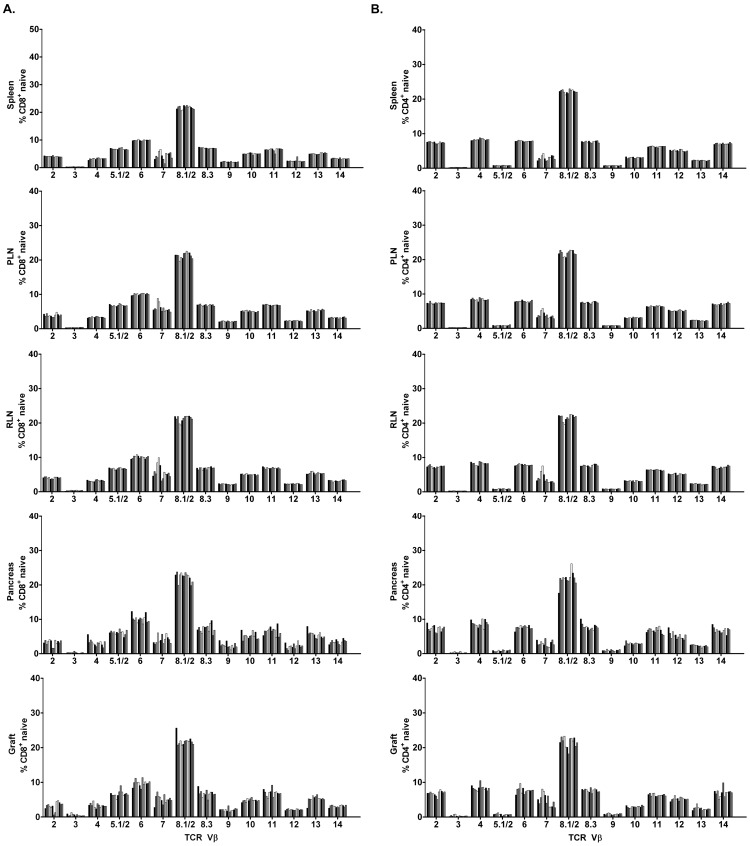
Analyses of TCR Vβ chain usage by naïve CD4^+^ and CD8^+^ T cells in islet graft NOD recipients. Frequency of TCR Vβ chains expressed by naïve CD8^+^ (A) and CD4^+^ (B) T cells in the spleen, PLN, RLN, pancreas, and islet graft of individual NOD recipients (n = 13).

TCR Vβ usage by naïve splenic CD8^+^ and CD4^+^ T cells, which represents the T cell repertoire under homeostasis, was essentially identical among the NOD recipients, and similar to the PLN and RLN (Figure 2). Interestingly, naïve CD8^+^ (Figure 2A) and CD4^+^ (Figure 2B) T cells found in the islet grafts and pancreas also exhibited a TCR Vβ repertoire analogous to the spleen. Naïve T cells were found at a relatively high frequency in the grafted (CD4^+^: 35.0±6.3%; CD8^+^: 25.7±6.3%) and endogenous (CD4^+^: 52.8±3.2%; CD8^+^: 40.3±4.0%) islets. These results demonstrate that TCR Vβ usage by naïve CD4^+^ and CD8^+^ T cells is diverse, with minimal (if any) variability among the tissues including grafted and endogenous islets in a given animal, and among NOD recipients.

### Islet Graft-infiltrating CD8^+^ Teff/mem Exhibit Restricted TCR Vβ Chain Usage

**Figure 3:**
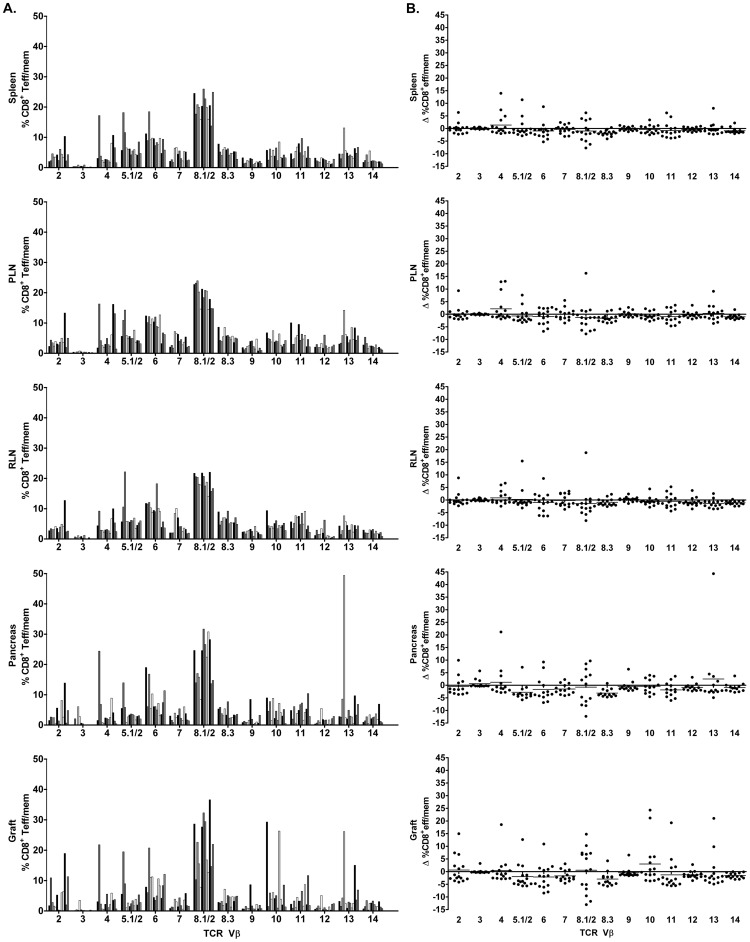
Analyses of TCR Vβ chain usage by CD8^+^ Teff/mem in islet graft NOD recipients. (A) Percentage of CD8^+^ Teff/mem expressing specific TCR Vβ chains in spleen, PLN, RLN, pancreas, and islet graft of individual NOD mice (n = 13). (B) The frequency of TCR Vβ chain usage by CD8^+^ Teff/mem was normalized by subtracting the frequency of the corresponding TCR Vβ chain expressed by splenic, naïve CD8^+^ T cells for a given NOD recipient.

In contrast to naïve CD8^+^ T cells, skewed TCR Vβ usage by CD8^+^ Teff/mem was observed in both the islet graft and pancreas of NOD recipients (Figure 3A; Tables 1, 2). Islet graft-infiltrating CD8^+^ Teff/mem preferentially expressed 1 to 4 TCR Vβ chains, making up>50% of the TCR Vβ repertoire in some mice (e.g. mouse #1, 2, 9, 11) (Table 1). Furthermore, TCR Vβ usage varied in the islet grafts among individual recipients which was readily evident: 1) by marked changes in the frequency of the majority of Vβ families (Figure 3A) compared to naïve CD8^+^ T cells found in the spleen or islet graft (Figure 2A), and 2) when TCR Vβ usage by CD8^+^ Teff/mem was normalized to splenic naïve CD8^+^ T cells (Figure 3B). Generally, the dominant TCR Vβ chains expressed by CD8^+^ Teff/mem infiltrating the graft were also prominent in the pancreas. For example, in mouse #2 the TCR Vβ repertoire of islet graft CD8^+^ Teff/mem was dominated by TCR Vβ4 (21.8%), Vβ5.1/2 (19.5%), Vβ2 (10.9%), and Vβ8.1/2 (10.3%) (Table 1); in the endogenous islets TCR Vβ4 (24.4%), Vβ5.1/2 (13.9%), and Vβ8.1/8.2 (13.9%) were also prevalent, although expression of TCR Vβ2 (2.1%) was limited (Table 2). These findings indicate that TCR Vβ usage by CD8^+^ Teff/mem is restricted in both the grafted and endogenous islets, and is variable among individual recipients.

**Table 1:** Frequency of TCR Vβ chains expressed by islet graft infiltrating CD8^+^ Teff/mem.

Vβ	Mouse1	Mouse2	Mouse3	Mouse4	Mouse5	Mouse6	Mouse7	Mouse8	Mouse9	Mouse10	Mouse11	Mouse12	Mouse13
**2**	1.8	10.9	2.8	1.6	1.3	5.3	0.3	0.2	6.0	6.3	19.0	2.0	11.3
**3**	0.1	0.2	0.4	0.2	3.5	0.4	0.2	0.2	0.0	0.0	0.1	0.3	0.0
**4**	3.1	21.8	2.3	0.7	0.5	2.2	5.6	1.1	2.1	5.9	3.5	3.8	0.7
**5.1/2**	5.6	19.5	8.9	1.1	2.6	1.5	2.4	3.4	1.7	3.9	2.0	5.3	2.8
**6**	7.9	6.0	20.7	11.1	11.2	4.5	3.5	5.0	10.6	8.3	4.2	8.4	12.1
**7**	0.8	1.3	1.1	3.9	3.0	1.4	4.3	1.8	0.6	3.6	5.8	1.7	1.5
**8.1/2**	28.6	10.3	22.6	15.5	7.8	27.7	32.3	29.4	16.8	12.7	36.6	14.6	21.9
**8.3**	2.9	2.5	3.2	1.9	7.2	2.5	5.1	4.7	1.3	4.7	3.2	4.7	4.9
**9**	0.7	0.6	1.5	0.9	0.7	8.7	1.7	0.7	0.6	2.2	0.7	1.9	0.8
**10**	29.3	1.5	5.9	3.9	6.1	4.1	5.1	1.0	26.3	3.9	2.0	8.5	1.4
**11**	2.3	1.0	3.3	1.1	4.5	3.3	3.3	6.5	1.6	8.7	2.1	11.7	1.8
**12**	0.6	1.0	1.7	0.9	5.1	0.4	1.1	0.4	0.2	1.3	0.3	2.2	2.4
**13**	3.2	1.6	4.3	26.1	2.5	0.9	3.0	2.6	1.0	2.6	15.0	3.6	6.9
**14**	0.7	1.5	3.5	0.3	2.5	2.6	3.3	2.1	1.4	1.4	3.1	1.3	0.8
**Nonreactive**	1.8	10.9	2.8	1.6	1.3	5.3	0.3	0.2	6.0	6.3	19.0	2.0	11.3

**Table 2:** Frequency of TCR Vβ chains expressed by pancreas infiltrating CD8^+^ Teff/mem.

Vβ	Mouse 1	Mouse 2	Mouse 3	Mouse 4	Mouse 5	Mouse 6	Mouse 7	Mouse 8	Mouse 9	Mouse 10	Mouse 11	Mouse 12	Mouse 13
**2**	1.5	2.7	2.4	2.4	0.6	5.6	1.4	0.2	8.1	2.6	13.9	0.7	4.8
**3**	0.0	2.1	0.0	6.0	2.8	0.6	0.4	0.1	0.1	0.1	0.0	0.1	0.0
**4**	1.5	24.4	6.8	0.8	0.6	2.4	2.2	1.9	2.5	8.9	4.1	1.3	0.4
**5.1/2**	5.5	13.9	5.8	0.9	2.7	3.4	3.7	3.5	3.1	1.7	2.9	3.1	2.0
**6**	19.0	6.1	16.8	5.5	10.3	6.1	6.1	5.0	7.2	3.1	3.5	7.4	11.4
**7**	3.0	1.6	0.8	4.0	2.5	3.2	5.4	2.2	1.5	6.0	3.8	1.7	1.4
**8.1/2**	24.7	13.9	17.0	15.8	8.5	24.6	31.7	26.6	22.4	30.8	28.2	13.7	14.8
**8.3**	5.3	5.9	3.8	3.1	5.3	3.3	7.7	2.3	2.4	2.5	3.4	3.1	3.6
**9**	0.8	1.3	0.9	0.8	1.6	8.5	1.9	0.3	0.6	1.0	0.6	3.2	0.0
**10**	9.0	4.6	7.8	1.5	8.8	2.2	4.6	1.6	7.2	4.1	3.0	5.1	0.5
**11**	4.8	2.0	6.1	0.6	3.9	4.9	6.7	7.3	1.6	4.1	5.3	10.4	2.9
**12**	0.4	0.7	1.5	0.8	5.5	1.8	1.5	1.7	0.3	1.7	0.3	2.1	2.9
**13**	2.8	2.6	8.6	50.4	2.4	2.0	4.9	3.0	2.7	0.5	9.7	3.3	6.8
**14**	1.0	1.4	2.7	0.5	3.4	2.1	2.4	2.7	3.7	1.9	6.9	1.2	0.8
**Nonreactive**	20.7	17.0	19.0	7.0	41.2	29.3	19.4	41.8	36.7	31.1	14.5	43.5	47.9

### Islet Graft-infiltrating, TCR Vβ8.1/2-expressing CD8^+^ Teff/mem exhibit IGRP_206–214_-specificity

**Figure 4:**
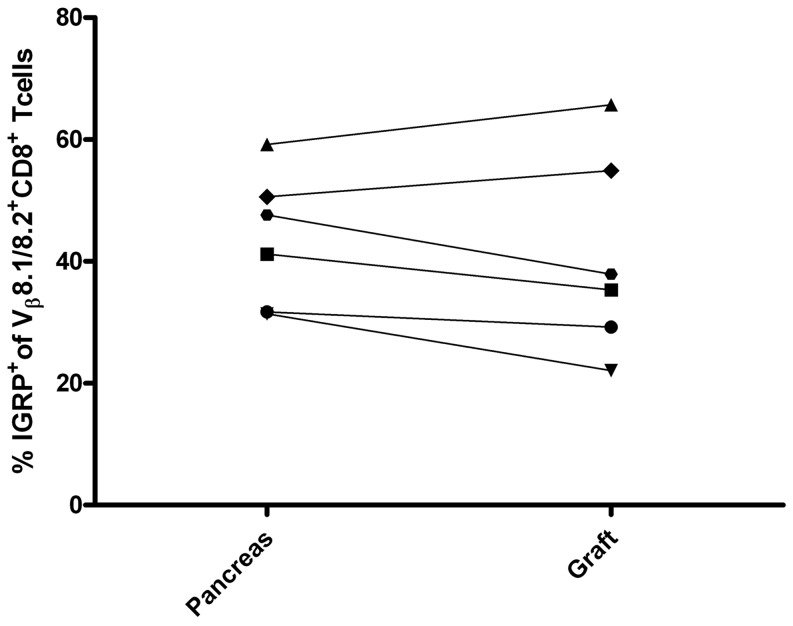
The frequency of H2K^d^-IGRP_206–214_ binding by TCR Vβ8.1/2-expressing CD8^+^ Teff/mem in grafted and endogenous islets in NOD recipients. The frequency of islet graft and pancreas-infiltrating TCR Vβ8.1/2 CD8^+^ T cells binding H2K^d^-IGRP_206–214_ tetramer was determined for an individual NOD recipient (n = 6).

The frequency of TCR Vβ8.1/2 expressing CD8^+^ Teff/mem infiltrating the grafted and endogenous islets was increased compared to splenic naïve CD8^+^ T cells in the majority (7/13) of NOD recipients (Figure 3B). We and others have shown that IGRP_206–214_-specific CD8^+^ T cells preferentially express TCR Vβ8.1/2, and are a dominant clonotype mediating β cell destruction in NOD mice [32]. Accordingly, the frequency of IGRP_206–214_-specific CD8^+^ T cells among TCR Vβ8.1/2-expressing CD8^+^ Teff/mem in the islet grafts (and pancreas) was determined using H2K^d^-IGRP tetramers. Strikingly, up to 65% of TCR Vβ8.1/2-expressing CD8^+^ Teff/mem in the grafted islets stained with the H2K^d^-IGRP (Figure 4). Interestingly, within a given recipient similar frequencies of IGRP_206–214_-specific CD8^+^ Teff/mem were detected in the grafted and endogenous islets (Figure 4). The increase in IGRP_206–214_-specific CD8^+^ Teff/mem within the pool of TCR Vβ8.1/2 expressing CD8^+^ Teff/mem was tissue-specific since H2K^d^-IGRP-binding TCR Vβ8.1/2-expressing CD8^+^ Teff/mem in the spleen was <0.05%. These results demonstrate that IGRP_206–214_-specific CD8^+^ Teff/mem make up a significant portion of the TCR Vβ8.1/2-expressing CD8^+^ Teff/mem that infiltrate both the grafted and endogenous islets.

### TCR Vβ12 Usage is Increased by Islet Graft-infiltrating CD4^+^ Teff/mem

**Figure 5:**
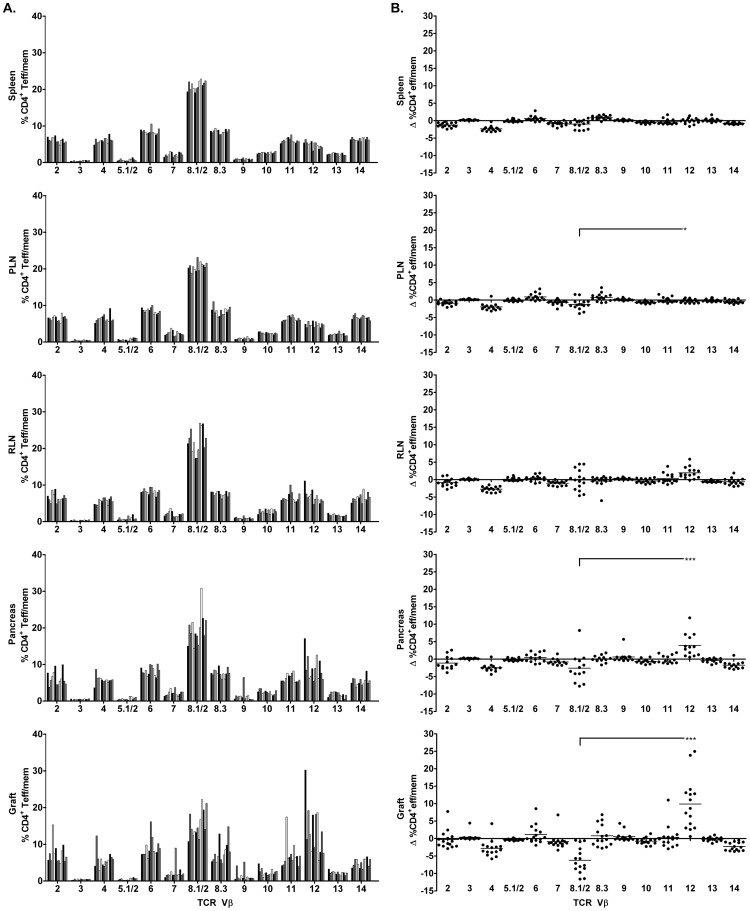
Analyses of TCR Vβ chain usage by CD4^+^ Teff/mem in islet graft NOD recipients. (A) Percentage of CD4^+^ Teff/mem expressing specific TCR Vβ chains in spleen, PLN, RLN, pancreas, and islet graft of individual NOD mice (n = 13). (B) TCR Vβ chain usage by CD4^+^ Teff/mem was normalized by subtracting the frequency of the corresponding TCR Vβ chain expressed by splenic, naïve CD4^+^ T cells for a given NOD recipient. ***p<0.001, *p<0.05; One way ANOVA with Dunn’s post-test.

**Figure 6:**
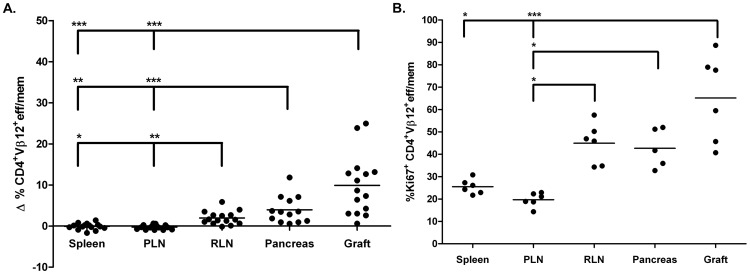
Tissue distribution and proliferation of TCR Vβ12-expressing CD4^+^ Teff/mem in NOD islet graft recipients. (A) Comparison among tissues of the change in frequency of TCR Vβ12-expressing CD4^+^ Teff/mem normalized by subtracting the percentage of TCR Vβ12-expressing splenic, naïve CD4^+^ T cells for a given NOD recipient (n = 13). (B) The tissue distribution of TCR Vβ12-expressing CD4^+^ Teff/mem staining for Ki67. ***p<0.001, **p<0.01, *p<0.05; Kruskal-Wallis test with two-sided Dunn's post-test.

Islet graft-infiltrating CD4^+^ Teff/mem were characterized by 1 to 4 prevalent TCR Vβ chains (Figure 5A; Table 3), similar to CD8^+^ Teff/mem. However, unlike CD8^+^ Teff/mem, the TCR Vβ repertoire expressed by CD4^+^ Teff/mem in the grafts of most NOD recipients (Figure 5A) generally resembled the diverse TCR Vβ profile used by naïve CD4^+^ T cells in the spleen (Figure 2B). The notable exception was TCR Vβ12 expression by CD4^+^ Teff/mem, which was increased in the islet grafts (and pancreas) of the majority of NOD recipients compared to naïve CD4^+^ T cells and CD4^+^ Teff/mem in the spleen (Figures 2B, 5A; Tables 2, 3). Skewing towards TCR Vβ12 usage by CD4^+^ Teff/mem in the islet graft and to a lesser degree in the pancreas was further evident when TCR Vβ usage within the respective tissues was normalized to the TCR repertoire of splenic naïve CD4^+^ T cells (Figure 5B). Normalized TCR Vβ12 usage by CD4^+^ Teff/mem was increased in grafted (10.0±7.3%) and endogenous (3.9±3.3%) islets relative to CD4^+^ Teff/mem found in the spleen (−0.1±0.8), RLN (2.0±1.6%) and/or PLN (−0.3±0.6%) (Figures 5B, 6A). While TCR Vβ12 did not dominate the CD4^+^ Teff/mem repertoire in all recipients, ranging between ∼5 to 30% of the total CD4^+^ Teff/mem population (Tables 3, 4), the largest percent increase was nevertheless detected for TCR Vβ12 in the islet graft and pancreas of the majority of recipients (Figures 5B, 6A). In contrast, despite being prevalent among all recipients, TCR Vβ8.1/2 usage by CD4^+^ Teff/mem in islet grafts and the pancreas was reduced relative to the TCR repertoire of naïve CD4^+^ T cells (Figure 5B).

**Table 3:** Frequency of TCR Vβ chains expressed by islet graft infiltrating CD4^+^ Teff/mem.

Vβ	Mouse 1	Mouse 2	Mouse 3	Mouse 4	Mouse 5	Mouse 6	Mouse 7	Mouse 8	Mouse 9	Mouse 10	Mouse 11	Mouse 12	Mouse 13
**2**	5.7	7.5	5.6	15.3	7.4	8.9	5.5	5.6	4.8	8.2	9.8	5.3	6.5
**3**	0.2	0.3	0.5	0.1	0.6	0.2	0.6	0.5	0.4	0.4	0.5	0.4	0.5
**4**	4.1	12.3	6.0	2.9	6.0	4.5	4.1	5.3	5.0	5.7	7.3	6.5	5.9
**5.1/2**	0.3	0.5	0.6	0.2	0.2	0.2	0.3	0.2	0.8	0.6	1.0	0.7	0.6
**6**	7.3	7.3	7.3	9.8	6.1	8.2	16.2	11.9	8.1	7.6	7.8	10.2	8.9
**7**	1.0	1.6	1.7	1.4	2.5	1.6	1.7	9.0	1.6	1.8	3.1	1.6	1.9
**8.1/2**	10.8	18.3	14.2	12.3	13.5	13.2	14.5	11.3	16.8	22.2	19.4	14.0	21.1
**8.3**	5.3	5.9	7.3	5.1	6.7	12.9	5.8	4.7	7.1	8.5	9.8	14.8	8.0
**9**	0.5	4.2	0.8	0.5	1.5	0.8	5.2	0.4	1.1	0.7	0.8	0.8	0.7
**10**	4.7	2.5	3.5	0.9	1.9	2.3	1.5	1.7	1.9	3.2	1.9	2.5	2.7
**11**	4.2	4.4	5.4	17.5	6.2	6.5	7.3	5.7	9.7	6.3	6.8	4.0	6.8
**12**	30.2	11.3	19.2	12.6	7.9	18.0	9.1	18.2	18.6	5.5	7.9	13.4	7.6
**13**	3.3	2.2	2.4	2.7	2.3	1.9	2.5	1.8	1.4	2.4	2.3	1.5	2.2
**14**	3.6	4.3	5.9	5.9	4.5	3.5	5.0	3.2	6.0	6.0	6.6	4.1	5.9
**Nonreactive**	5.7	7.5	5.6	15.3	7.4	8.9	5.5	5.6	4.8	8.2	9.8	5.3	6.5

**Table 4:** Frequency of TCR Vβ chains expressed by pancreas infiltrating CD4^+^ Teff/mem.

Vβ	Mouse 1	Mouse 2	Mouse 3	Mouse 4	Mouse 5	Mouse 6	Mouse 7	Mouse 8	Mouse 9	Mouse 10	Mouse 11	Mouse 12	Mouse 13
**2**	7.7	3.8	5.7	6.7	7.8	9.6	4.5	4.5	5.5	6.1	9.9	5.4	4.7
**3**	0.6	0.1	0.5	0.4	0.4	0.5	0.5	0.4	0.4	0.6	0.5	0.5	0.6
**4**	3.6	8.7	6.3	6.3	6.2	5.8	5.3	5.5	5.8	5.1	5.6	5.6	5.8
**5.1/2**	0.3	0.5	0.4	0.7	0.4	0.4	0.4	0.2	1.3	1.3	0.4	0.9	1.0
**6**	9.1	7.8	7.5	8.4	6.7	7.4	10.0	9.8	8.9	7.1	6.4	10.1	8.4
**7**	1.3	1.6	1.7	2.5	3.5	2.1	2.2	3.7	1.7	1.3	1.9	2.5	2.5
**8.1/2**	15.0	20.8	18.5	21.5	14.0	18.4	17.8	15.2	20.1	30.9	22.6	17.9	22.0
**8.3**	7.6	7.2	8.5	8.3	7.6	9.7	7.3	6.0	7.5	6.0	7.4	9.3	7.6
**9**	0.6	1.4	1.1	1.3	1.4	1.0	6.5	0.6	1.3	1.5	0.5	0.4	0.4
**10**	2.6	3.4	3.4	2.2	2.3	2.8	2.5	2.1	2.6	1.7	1.5	1.8	2.9
**11**	5.5	5.5	5.2	6.2	7.6	6.9	6.8	7.6	8.2	5.0	5.3	5.3	5.8
**12**	17.1	8.5	12.2	6.2	6.8	8.9	5.4	8.9	12.6	6.2	11.0	7.6	5.9
**13**	1.0	1.8	2.5	2.3	2.5	2.5	2.3	2.3	1.6	1.3	1.8	0.6	1.6
**14**	4.9	6.2	6.1	3.8	4.5	4.8	6.0	4.2	4.7	5.7	8.2	4.9	5.6
**Nonreactive**	7.7	3.8	5.7	6.7	7.8	9.6	4.5	4.5	5.5	6.1	9.9	5.4	4.7

The frequency of Ki67-staining CD4^+^ and CD8^+^ Teff/mem was determined in the spleen, islet graft and pancreas of individual NOD recipients.

*** p<0.001,

** p<0.01; Student’s t test.

The marked increase in islet graft-infiltrating TCR Vβ12-expressing CD4^+^ Teff/mem may be explained by tissue-specific expansion. To address this possibility, the proliferative status of TCR Vβ12-expressing CD4^+^ Teff/mem was examined by measuring Ki67 expression [40], [41]. The majority (up to 88%) of TCR Vβ12-expressing CD4^+^ Teff/mem in islet grafts were Ki67^+^ (Figure 6B). The frequency of Ki67^+^ TCR Vβ12-expressing CD4^+^ Teff/mem was significantly increased (∼2.5-fold) in the islets, and to a lesser extent in the pancreas, compared to the corresponding splenic CD4^+^ Teff/mem (Figure 6B). Interestingly, the frequency of Ki67^+^ TCR Vβ12-expressing CD4^+^ Teff/mem was also increased in the draining RLN compared to the spleen (Figure 6B). These findings indicate that in most NOD recipients the TCR Vβ repertoire in islet grafts is characterized by an increase in TCR Vβ12 usage, which in turn is associated with elevated Vβ12-specific CD4^+^ Teff/mem proliferation.

### CD8^+^ Teff/mem Exhibit Limited TCR Vβ Chain Diversity in Grafted and Endogenous Islets

**Figure 7:**
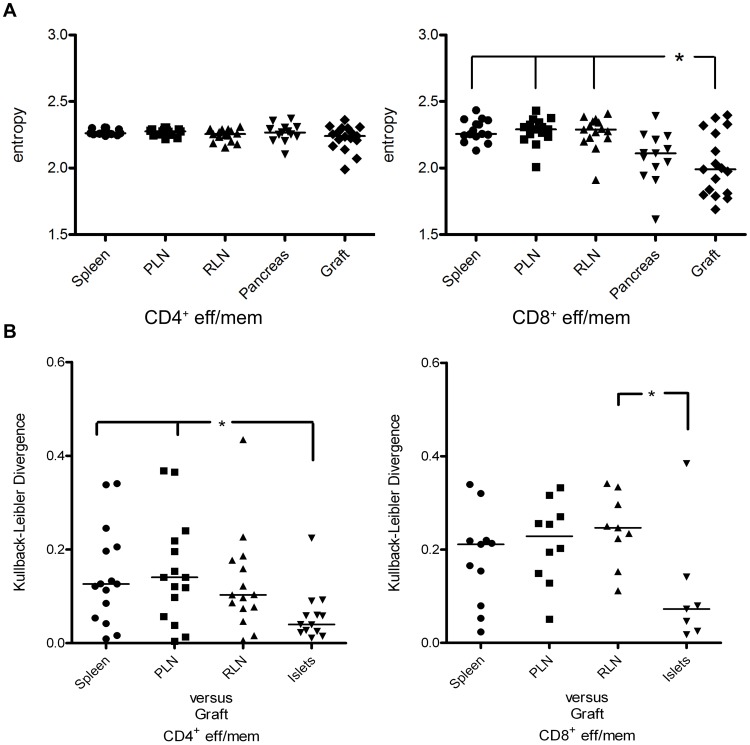
Tissue-specific TCR Vβ chain diversity and distribution for CD4^+^ and CD8^+^ Teff/mem in NOD islet graft recipients. The diversity (A) and distribution (B) of Vβ chain usage by tissue-specific CD4^+^ and CD8+ Teff/mem in NOD islet graft recipients (n = 13) was determined by Shannon entropy and Kullback-Leibler divergence, respectively. For the latter, the X axis represents comparisons of the respective tissues to the islet graft of a given NOD recipient. *p<0.05; Kruskal-Wallis test with two-sided Dunn's post-test.

To more accurately assess the level of diversity among TCR Vβ chains expressed by naïve T cells and Teff/mem in the respective tissues, the Shannon entropy was calculated. The Shannon entropy within a given population represents both the species richness (number of Vβ chains expressed) and relative abundance (proportion of each Vβ chain) in the sample [42]. Hence, if a pool of T cells expresses a small number TCR Vβ chain families that are prevalent, entropy will be correspondingly reduced relative to a TCR repertoire consisting of several evenly distributed Vβ chain families. As expected, the entropy of naïve CD4^+^ and CD8^+^ T cells was nearly identical for all tissues (data not shown), indicating a similar level of TCR Vβ diversity. In contrast, the entropy for TCR Vβ chains of CD8^+^ Teff/mem was reduced in the pancreas, and significantly lower in islet grafts compared to spleen, PLN and RLN (Figure 7A), thereby indicating restricted TCR Vβ usage. On the other hand, entropy of TCR Vβ chains expressed by CD4^+^ Teff/mem in grafted and endogenous islets was similar to that detected for spleen, PLN and RLN (Figure 7A). Furthermore, entropy for islet graft-infiltrating CD4^+^ (2.23±0.02) versus CD8^+^ (2.03±0.02) Teff/mem was significantly increased (p = 0.03; Mann-Whitney U). These data demonstrate that CD8^+^ Teff/mem in the islet graft, and to a lesser extent the pancreas, have significantly restricted TCR Vβ chain usage in comparison to CD8^+^ Teff/mem in spleen and lymph nodes. The TCR repertoire of CD4^+^ Teff/mem in the grafted (and endogenous) islets, however, is more diverse than that of CD8^+^ Teff/mem.

### Similar TCR Vβ Chain Repertoires are Expressed by Teff/mem Infiltrating the Islet Graft and Pancreas

As noted earlier, TCR Vβ usage by CD4^+^ and CD8^+^ Teff/mem was similar between the islet graft and pancreas in individual recipients (Figures 3, 5; Tables 1,2,3,4). The Kullback-Liebler divergence was employed to quantify the level of similarity of TCR Vβ usage among CD4^+^ and CD8^+^ naïve and Teff/mem in the respective tissues. The Kullback-Liebler divergence is a measure of the difference between two distributions, and as such allows for the comparison of Vβ usage similarities between tissues [43]–[45]; divergence is low for instance if two Vβ distributions are similar. TCR Vβ usage among naïve CD4^+^ and CD8^+^ T cells was similar for the respective tissues, with a divergence index of approximately 0.0 (data not shown). Notably, the average divergence index detected for CD8^+^ Teff/mem in the islet graft versus pancreas (0.11±0.05) was reduced relative to comparisons between the islet graft and the spleen (0.18±0.03), PLN (0.22±0.03), and RLN (0.24±0.03) (Figure 7B). Similar trends were observed for islet graft-infiltrating CD4^+^ Teff/mem where the divergence index for the TCR repertoire between the islet graft and pancreas (0.059±0.02) was less than the divergence index calculated for comparisons between the islet graft versus spleen (0.14±0.03), PLN (0.15±0.03) and RLN (0.13±0.03) (Figure 7B). These results demonstrate that the distribution of Vβ usage by CD4^+^ and CD8^+^ Teff/mem in grafted islets is more similar to Vβ usage in the endogenous islets than that in the spleen, PLN and RLN.

## Discussion

The TCR Vβ repertoire of islet-infiltrating T cells has been investigated mostly by RT-PCR from bulk RNA isolated from the islets or via flow cytometric-sorted MHC multimer-binding T cells [20], [21], [32], [42], [46]–[56]. Studies using the former approach typically have not distinguished between naïve and eff/mem T cells. The use of MHC multimer-sorted T cells is also limited since information is obtained for clonotypes specific only for a given peptide epitope. To overcome these limitations, a novel multi-parameter flow cytometry approach was employed to characterize the TCR Vβ repertoire of CD4^+^ and CD8^+^ T cells involved in autoimmune destruction of islet grafts in individual NOD recipients. The approach permits analyses of general shifts in TCR usage in a tissue-specific manner while taking in consideration the activation status of the T cells. Exploiting this strategy, 3 key observations were made. First, naïve CD4^+^ and CD8^+^ T cells make up a significant frequency of islet graft-infiltrating T cells, which express a TCR repertoire analogous to naïve T cells in peripheral lymphoid organs and endogenous islets. Second, TCR Vβ usage by islet graft-infiltrating Teff/mem is restricted, with the TCR repertoire of CD8^+^ versus CD4^+^ Teff/mem exhibiting distinct characteristics. Third, the TCR Vβ repertoire is similar for CD8^+^ and CD4^+^ Teff/mem infiltrating grafted and endogenous islets of a given NOD recipient.

Our group has previously reported a high frequency of naïve T cells infiltrating the pancreas of NOD mice [57]. A similarly high percentage of naïve T cells was also detected in grafted islets. Currently there is controversy regarding the need for antigen stimulation of β cell-specific T cells to traffick into the islets [47], [58]–[60]. The fact that naïve CD4^+^ and CD8^+^ T cells are found at a relatively high frequency argues against antigen-specificity/stimulation playing an essential role for T cell trafficking into grafted and endogenous islets. This scenario is further supported by our observation that TCR Vβ usage by naïve T cells in the islet graft and pancreas is identical to the spleen, PLN and RLN (Figure 2), and lacks the restriction seen by antigen-stimulated Teff/mem. Ongoing inflammation and production of chemokines for example, likely provide signals that promote trafficking of naïve T cells into the respective tissues. Indeed, naïve T cells are readily detected in islet grafts at day 10 post-implantation but comprise only a small percentage of T cells 5 days after transplantation (unpublished results; R.D., A.G, R.T.). Our findings also underscore the need to define the activational status of T cells in order to accurately determine potential shifts in TCR repertoire. For example, a predominant pool of naïve T cells may obscure subtle but significant changes in TCR usage by disease-relevant Teff/mem.

We have reported earlier that islet graft-infiltrating IGRP_206–214_-specific CD8^+^ T cells exhibit a skewed repertoire consisting of 1 to 3 clones based on TCR Vβ CDR3 sequences [32]. Analyses of TCR Vβ usage carried out in the current study indicate that a highly restricted TCR repertoire is in fact a general property of CD8^+^ Teff/mem infiltrating an islet graft and the pancreas. Islet graft-infiltrating CD8^+^ Teff/mem expressed 1 to 4 prevalent TCR Vβ chains in a given NOD recipient (Figure 3; Table 1), which in turn was demonstrated by reduced entropy relative to CD8^+^ Teff/mem found in the spleen, PLN and RLN of recipient NOD mice (Figure 7A). Furthermore, TCR Vβ usage by islet graft-infiltrating CD8^+^ Teff/mem varied markedly among NOD recipients (Figure 3B,D; Table 1), again consistent with our earlier observations made for IGRP_206–214_-specific CD8^+^ T cells [32]. Noteworthy is that up to 65% of islet graft-infiltrating CD8^+^ T cells expressing TCR Vβ8.1/2 were IGRP_206–214_-specific (Figure 4). Whether the different TCR Vβ chains used by CD8^+^ Teff/mem among individual NOD recipients is due to recognition of the same autoantigen by distinct clones, and/or multiple autoantigens and/or epitopes remains to be determined.

Analogous to CD8^+^ Teff/mem, islet graft-infiltrating CD4^+^ Teff/mem were characterized by expression of a limited number of prevalent TCR Vβ chains (Figure 4; Table 3). Nevertheless, TCR Vβ usage by CD4^+^ Teff/mem exhibited characteristics distinct from CD8^+^ Teff/mem. The most striking difference was the increased usage of TCR Vβ12 by islet graft-infiltrating CD4^+^ Teff/mem in the majority (12/13) of NOD recipients (Figure 5; Table 3). Skewed TCR Vβ12 usage by CD4^+^ T cells in the pancreas of prediabetic NOD mice has previously been reported [19], [49], [56], [61]. In view of the high frequency of H2K^d^-IGRP_206–214_ binding among TCR Vβ8.1/2-expressing CD8^+^ Teff/mem (Figure 4), it is tempting to speculate that TCR Vβ12-expressing CD4^+^ Teff/mem also represent a set of clones targeting a particular β cell autoantigen [61]. Interestingly, TCR Vβ12 expression appears to be primarily associated with pathogenic CD4^+^ Teff/mem since islet graft and pancreas-infiltrating Foxp3^+^CD4^+^ T cells do not exhibit the same increase of TCR Vβ12 usage within and among the individual NOD recipients (unpublished results; R.D., A.G., and R.T.). TCR Vβ usage by islet graft (and pancreas)-infiltrating CD4^+^ Teff/mem also differed in terms of the extent of overall diversity compared to CD8^+^ Teff/mem. Despite increased usage of certain TCR Vβ chains (e.g. Vβ12), islet graft-infiltrating CD4^+^ Teff/mem also expressed other Vβ chains at frequencies similar to naïve CD4^+^ T cells residing in the spleen (Figures 2, 5; Table 3). For instance, in the islet graft of mouse #1 in which TCR Vβ12 (30.2%), and Vβ8.1/2 (10.8%) were markedly increased, other prevalent TCR Vβ chains were used by CD4^+^ Teff/mem including Vβ2 (5.7%), Vβ4 (4.1%), Vβ6 (7.3%), Vβ8.3 (5.3%), Vβ10 (4.7%), and Vβ11 (4.2%) (Table 3). Consequently, TCR Vβ diversity within islet graft and pancreas-infiltrating CD4^+^ versus CD8^+^ Teff/mem was increased (Figures 3A, 5A), which was evident by no significant change in Shannon entropy for CD4^+^ Teff/mem between tissues (Figure 7A). The more diverse TCR Vβ repertoire may indicate a broader range of β cell autoantigens targeted in the islet grafts (and pancreas) by CD4^+^ versus CD8^+^ Teff/mem.

Immunodominance of particular TCR Vβ-expressing Teff/mem is expected to be attributed to clonal expansion within the islet grafts. Indeed, evidence indicates that increased TCR Vβ chain usage is associated with elevated proliferation of Teff/mem residing in the islet graft (and pancreas). For instance, TCR Vβ12-expressing CD4^+^ Teff/mem found in the grafted and endogenous islets displayed increased proliferation relative to the PLN and RLN (and spleen) (Figure 6B), consistent with antigen-driven expansion. In addition, ∼70% of islet graft-infiltrating CD8^+^ Teff/mem were proliferating based on Ki67-staining (Figure S1). Interestingly, the frequency of proliferating CD4^+^ Teff/mem was ∼2-fold less (Figure S1), which may partly explain the greater skewing of TCR usage seen by CD8^+^ versus CD4^+^ Teff/mem in the islet grafts (Figures 3A, 5A). In contrast, a diverse TCR repertoire was associated with the nonproliferating, naïve CD8^+^ and CD4^+^ T cells found in the islet graft and pancreas.

Another key observation made in this study is that the TCR Vβ repertoire of islet graft-infiltrating CD4^+^ and CD8^+^ Teff/mem was more similar to the repertoire of Teff/mem residing in the pancreas than that detected in the PLN, RLN, and spleen (Figure 7B). In addition, similarities in TCR repertoires between the 2 sites was evident by equivalent frequencies of IGRP_206–214_-specific CD8^+^ Teff/mem among TCR Vβ8.1/2 expressing cells in grafted and endogenous islets (Figure 4). Increased frequencies of proliferating TCR Vβ12-expressing CD4^+^ Teff/mem were also observed in both the grafted and endogenous islets of NOD recipients (Figure 6B). This data supports a scenario in which β cell-specific T cells mediating recurrent autoimmunity are recruited from a pool of CD4^+^ and CD8^+^ Teff/mem involved in endogenous islet destruction, rather than from naïve β cell-specific clonotypes found in the periphery. The rapid kinetics of syngeneic islet graft rejection, generally seen within 10–14 days post-implantation, is consistent with the recruitment of established β cell-specific Teff/mem. Additional studies are needed to directly define the specificity of Teff/mem residing in the grafted and endogenous islets to confirm our model.

In conclusion, our findings demonstrate that autoreactive Teff/mem driving islet graft rejection express a restricted TCR repertoire resembling that of Teff/mem involved in the destruction of endogenous islets. Notably, the nature of the TCR repertoire regarding TCR Vβ usage and the extent of diversity differ between CD4^+^ and CD8^+^ Teff/mem infiltrating the islet grafts. These findings provide new insight into the dynamics and scope of the autoimmune TCR Vβrepertoire of CD4^+^ and CD8^+^ T cells, which may aid in the development of biomarkers and more effective immunotherapies to monitor and block islet graft rejection. Indeed, it is noteworthy that a recent study has shown that treatment with an anti-TCR Vβ13 antibody effectively prevents diabetes in the diabetes-prone BioBreeding rat model of T1D [63].

## Materials and Methods

### Mice

NOD/LtJ and NOD.CB17.Prkdc*scid*/J (NOD.*scid*) mice were bred and housed under pathogen-free conditions in an American Association for Laboratory-accredited animal facility. NOD mice were considered to be diabetic after 2 successive days of ≥250 mg/dl blood glucose as measured by a Freestyle *Lite* blood glucose monitor and strips (Abbott Diabetes Care Inc.). All procedures were reviewed and approved by the University of North Carolina Institutional Animal Care and Use Committee.

### Islet Transplantation

Diabetic NOD female mice received 5 units of insulin daily prior to transplantation. Five hundred syngeneic (NOD.*scid*) islets were transplanted under the renal capsule of the left kidney. Blood glucose values were monitored daily post-transplantation.

### Flow Cytometry

Spleen, PLN, RLN, and pancreas single-cell suspensions were prepared by grinding tissue between frosted slides in RPMI complete containing 100 nM Dasatinib, which not only inhibits downregulation of the TCR but it also increases TCR and co-receptor surface expression allowing for better staining [62]. When required, red cells were lysed with RBC lysis buffer. Islet grafts were excised from the kidney and gently ground to release infiltrating cells under the capsule and minimize kidney cell contamination. Cells were washed with FACS buffer (PBS plus 0.5% BSA), filtered and blocked with αCD16/32 (2.4G2). Cells were always kept in media containing 100 nM Dasatinib. Samples were then split into three wells and stained. Thy1.1^+^ spleen cells (1×10^6^) were added to wells containing cells from grafts and PLN/RLN prior to addition of antibodies as a staining internal control. Cells were stained with antibodies specific for CD90.2 (53–2.1), CD8 (53–6.7), CD44 (IM7), CD62L (MEL-14) (BD Biosciences), Thy1.1 (OX-7) (BioLegend), CD4^+^ (RM4-5) (Invitrogen) and three different anti-TCR Vβ panels. **Panel A:** αTCR Vβ2-biotin (B20.6), αTCR Vβ3-PE (KJ25), αTCR Vβ4-biotin (KT4), αTCR Vβ4-FITC, αTCR Vβ6-biotin (RRA-7), αTCR Vβ6-PE and αTCR Vβ9-FITC (MR10-2); **Panel B:** αTCR Vβ5.1/2-biotin (MR9-4), αTCR Vβ7-PE (TR310), αTCR Vβ8.1/2-FITC (MR5-2), αTCR Vβ 8.1/2-biotin and αTCR Vβ8.3-FITC (1B3.3); **Panel C:** αTCR Vβ10[b]-FITC (B21.5), αTCR Vβ 10[b]-PE, αTCR Vβ11-PE (RR3-15), αTCR Vβ11-biotin, αTCR Vβ12-biotin (MR11-1), αTCR Vβ13-PE (MR12-4) (Biolegend) and αTCR Vβ14-FITC (14–2). All αTCR Vβ antibodies were purchased from BD Biosciences unless noted. Binding of biotin-labeled antibodies was revealed by streptavidin Alexa 594 (Invitrogen). Cells were washed twice with PBS and stained with LIVE/DEAD® Fixable Blue Dead Cell Stain Kit (Invitrogen) to exclude dead cells. To stain for FoxP3 (FJK-16s) or Ki67 (B56) samples were washed, fixed and permeabilized with eBiosciences Fix/Perm kit following manufacturer’s indications. For tetramer analysis, H2K^d^-IGRP_206–214_ tetramers were prepared as previously described [32]. Cells were first stained with H2K^d^-IGRP_206–214_ for 40 minutes at room temperature, and then placed on ice and incubated for 20 minutes with 100 µL of a 2X cocktail of antibodies specific for T cell markers and TCR Vβ chains. Data was acquired at the University of North Carolina Flow Cytometry Facility using a 6 laser (355 nm, 405 nm, 488 nm, 532 nm, 592 nm and 640 nm), 18 parameter LSRII Special Order Research Product flow cytometer (BD Biosciences). Analysis was performed with FlowJo software (Tree Star Inc.).

### Diversity and Distribution Analyses

Shannon entropy was used as an index of TCR Vβ usage diversity. As described by Vincent *et al* [42], the entropy of a Vβ usage distribution is determined by two parameters: 1) the number of different Vβ chains that are expressed, and 2) the relative frequency of each individual Vβ chain. Entropy is greatest when there are many different Vβs and when there are few Vβ chains that are highly represented in the population (i.e. few “dominant” families). If *S* is the total number of unique Vβ chains in the pool, and *p_i_* is the proportion of the pool represented by Vβ *i*, the Shannon entropy *H* is defined as:
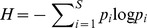



The Kullback-Leibler^34^ divergence was used as an index of similarity between TCR Vβ usage distributions and is defined as:
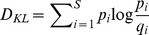
where *p_i_* is the proportion of the pool represented by Vβ *i* in the first sample and *q_i_* is the proportion of that Vβ in the second sample.

## Supporting Information

Figure S1 Increased proliferation of islet graft-infiltrating Teff/mem in individual NOD recipients. The frequency of Ki67-staining CD4^+^ and CD8^+^ Teff/mem was determined in the spleen, islet graft and pancreas of individual NOD recipients. ***p<0.001, **p<0.01; Student’s t test.
(TIFF)

## References

[1] Anderson MS, Bluestone JA (2005) The NOD mouse: a model of immune dysregulation. Annu Rev Immunol 23: 447–485. 15771578 10.1146/annurev.immunol.23.021704.115643

[2] Bach JF (1994) Insulin-dependent diabetes mellitus as an autoimmune disease. Endocr Rev 15: 516–542. 7988484 10.1210/edrv-15-4-516

[3] Bluestone JA, Herold K, Eisenbarth G (2010) Genetics, pathogenesis and clinical interventions in type 1 diabetes. Nature 464: 1293–1300. 20432533 10.1038/nature08933 PMC4959889

[4] Tisch R, McDevitt H (1996) Insulin-dependent diabetes mellitus. Cell 85: 291–297. 8616883 10.1016/s0092-8674(00)81106-x

[5] van Belle TL, Coppieters KT, von Herrath MG (2011) Type 1 diabetes: etiology, immunology, and therapeutic strategies. Physiol Rev 91: 79–118. 21248163 10.1152/physrev.00003.2010

[6] Delovitch TL, Singh B (1997) The nonobese diabetic mouse as a model of autoimmune diabetes: immune dysregulation gets the NOD. Immunity 7: 727–738. 9430219 10.1016/s1074-7613(00)80392-1

[7] Mallone R, van Endert P (2008) T cells in the pathogenesis of type 1 diabetes. Curr Diab Rep 8: 101–106. 18445351 10.1007/s11892-008-0019-9

[8] Serreze DV, Leiter EH, Christianson GJ, Greiner D, Roopenian DC (1994) Major histocompatibility complex class I-deficient NOD-B2mnull mice are diabetes and insulitis resistant. Diabetes 43: 505–509. 8314025 10.2337/diab.43.3.505

[9] Shizuru JA, Taylor-Edwards C, Banks BA, Gregory AK, Fathman CG (1988) Immunotherapy of the nonobese diabetic mouse: treatment with an antibody to T-helper lymphocytes. Science 240: 659–662. 2966437 10.1126/science.2966437

[10] Wang B, Gonzalez A, Benoist C, Mathis D (1996) The role of CD8+ T cells in the initiation of insulin-dependent diabetes mellitus. Eur J Immunol 26: 1762–1769. 8765018 10.1002/eji.1830260815

[11] Brooks-Worrell B, Gersuk VH, Greenbaum C, Palmer JP (2001) Intermolecular antigen spreading occurs during the preclinical period of human type 1 diabetes. J Immunol 166: 5265–5270. 11290812 10.4049/jimmunol.166.8.5265

[12] Di Lorenzo TP, Peakman M, Roep BO (2007) Translational mini-review series on type 1 diabetes: Systematic analysis of T cell epitopes in autoimmune diabetes. Clin Exp Immunol 148: 1–16. 17349009 10.1111/j.1365-2249.2006.03244.x PMC1868845

[13] Kaufman DL, Clare-Salzler M, Tian J, Forsthuber T, Ting GS, et al (1993) Spontaneous loss of T-cell tolerance to glutamic acid decarboxylase in murine insulin-dependent diabetes. Nature 366: 69–72. 7694152 10.1038/366069a0 PMC8216222

[14] Li L, Wang B, Frelinger JA, Tisch R (2008) T-cell promiscuity in autoimmune diabetes. Diabetes 57: 2099–2106. 18492786 10.2337/db08-0383 PMC2494676

[15] Prasad S, Kohm AP, McMahon JS, Luo X, Miller SD (2012) Pathogenesis of NOD diabetes is initiated by reactivity to the insulin B chain 9–23 epitope and involves functional epitope spreading. J Autoimmun. 10.1016/j.jaut.2012.04.005 PMC3434243 22647732

[16] Tian J, Gregori S, Adorini L, Kaufman DL (2001) The frequency of high avidity T cells determines the hierarchy of determinant spreading. J Immunol 166: 7144–7150. 11390460 10.4049/jimmunol.166.12.7144

[17] Tisch R, Yang XD, Singer SM, Liblau RS, Fugger L, et al (1993) Immune response to glutamic acid decarboxylase correlates with insulitis in non-obese diabetic mice. Nature 366: 72–75. 8232539 10.1038/366072a0

[18] Zechel MA, Krawetz MD, Singh B (1998) Epitope dominance: evidence for reciprocal determinant spreading to glutamic acid decarboxylase in non-obese diabetic mice. Immunol Rev 164: 111–118. 9795769 10.1111/j.1600-065x.1998.tb01213.x

[19] Nakano N, Kikutani H, Nishimoto H, Kishimoto T (1991) T cell receptor V gene usage of islet beta cell-reactive T cells is not restricted in non-obese diabetic mice. J Exp Med 173: 1091–1097. 1902501 10.1084/jem.173.5.1091 PMC2118862

[20] Toyoda H, Redford A, Magalong D, Chan E, Hosszufalusi N, et al (1992) In situ islet T cell receptor variable region gene usage in the nonobese diabetic mouse. Immunol Lett 32: 241–245. 1379982 10.1016/0165-2478(92)90056-t

[21] Waters SH, O'Neil JJ, Melican DT, Appel MC (1992) Multiple TCR V beta usage by infiltrates of young NOD mouse islets of Langerhans. A polymerase chain reaction analysis. Diabetes 41: 308–312. 1372574 10.2337/diab.41.3.308

[22] Candeias S, Katz J, Benoist C, Mathis D, Haskins K (1991) Islet-specific T-cell clones from nonobese diabetic mice express heterogeneous T-cell receptors. Proc Natl Acad Sci U S A 88: 6167–6170. 2068098 10.1073/pnas.88.14.6167 PMC52043

[23] DiLorenzo TP, Graser RT, Ono T, Christianson GJ, Chapman HD, et al (1998) Major histocompatibility complex class I-restricted T cells are required for all but the end stages of diabetes development in nonobese diabetic mice and use a prevalent T cell receptor alpha chain gene rearrangement. Proc Natl Acad Sci U S A 95: 12538–12543. 9770521 10.1073/pnas.95.21.12538 PMC22866

[24] Haskins K, Portas M, Bergman B, Lafferty K, Bradley B (1989) Pancreatic islet-specific T-cell clones from nonobese diabetic mice. Proc Natl Acad Sci U S A 86: 8000–8004. 2510155 10.1073/pnas.86.20.8000 PMC298201

[25] Nagata M, Yoon JW (1992) Studies on autoimmunity for T-cell-mediated beta-cell destruction. Distinct difference in beta-cell destruction between CD4+ and CD8+ T-cell clones derived from lymphocytes infiltrating the islets of NOD mice. Diabetes 41: 998–1008. 1628775 10.2337/diab.41.8.998

[26] Wegmann DR, Norbury-Glaser M, Daniel D (1994) Insulin-specific T cells are a predominant component of islet infiltrates in pre-diabetic NOD mice. Eur J Immunol 24: 1853–1857. 8056042 10.1002/eji.1830240820

[27] Plesner A, Verchere CB (2011) Advances and challenges in islet transplantation: islet procurement rates and lessons learned from suboptimal islet transplantation. J Transplant 2011: 979527. 22235361 10.1155/2011/979527 PMC3253477

[28] Ryan EA, Paty BW, Senior PA, Bigam D, Alfadhli E, et al (2005) Five-year follow-up after clinical islet transplantation. Diabetes 54: 2060–2069. 15983207 10.2337/diabetes.54.7.2060

[29] Shapiro AM, Ricordi C, Hering BJ, Auchincloss H, Lindblad R, et al (2006) International trial of the Edmonton protocol for islet transplantation. N Engl J Med 355: 1318–1330. 17005949 10.1056/NEJMoa061267

[30] Coulombe M, Gill RG (2004) The immunobiology of pancreatic islet transplantation. Adv Exp Med Biol 552: 154–169. 15622963

[31] Wicker LS, Leiter EH, Todd JA, Renjilian RJ, Peterson E, et al (1994) Beta 2-microglobulin-deficient NOD mice do not develop insulitis or diabetes. Diabetes 43: 500–504. 8314024 10.2337/diab.43.3.500

[32] Wong CP, Li L, Frelinger JA, Tisch R (2006) Early autoimmune destruction of islet grafts is associated with a restricted repertoire of IGRP-specific CD8+ T cells in diabetic nonobese diabetic mice. J Immunol 176: 1637–1644. 16424193 10.4049/jimmunol.176.3.1637

[33] Jager E, Maeurer M, Hohn H, Karbach J, Jager D, et al (2000) Clonal expansion of Melan A-specific cytotoxic T lymphocytes in a melanoma patient responding to continued immunization with melanoma-associated peptides. Int J Cancer 86: 538–547. 10797269 10.1002/(sici)1097-0215(20000515)86:4<538::aid-ijc16>3.0.co;2-g

[34] Li J, Sze DM, Brown RD, Cowley MJ, Kaplan W, et al (2010) Clonal expansions of cytotoxic T cells exist in the blood of patients with Waldenstrom macroglobulinemia but exhibit anergic properties and are eliminated by nucleoside analogue therapy. Blood 115: 3580–3588. 20190191 10.1182/blood-2009-10-246991

[35] Morice WG, Kimlinger T, Katzmann JA, Lust JA, Heimgartner PJ, et al (2004) Flow cytometric assessment of TCR-Vbeta expression in the evaluation of peripheral blood involvement by T-cell lymphoproliferative disorders: a comparison with conventional T-cell immunophenotyping and molecular genetic techniques. Am J Clin Pathol 121: 373–383. 15023042 10.1309/3A32-DTVM-H640-M2QA

[36] Pilch H, Hohn H, Freitag K, Neukirch C, Necker A, et al (2002) Improved assessment of T-cell receptor (TCR) VB repertoire in clinical specimens: combination of TCR-CDR3 spectratyping with flow cytometry-based TCR VB frequency analysis. Clin Diagn Lab Immunol 9: 257–266. 11874861 10.1128/CDLI.9.2.257-266.2002 PMC119929

[37] Berard M, Tough DF (2002) Qualitative differences between naive and memory T cells. Immunology 106: 127–138. 12047742 10.1046/j.1365-2567.2002.01447.x PMC1782715

[38] Budd RC, Cerottini JC, MacDonald HR (1987) Selectively increased production of interferon-gamma by subsets of Lyt-2+ and L3T4+ T cells identified by expression of Pgp-1. J Immunol 138: 3583–3586. 3108367

[39] DeGrendele HC, Estess P, Siegelman MH (1997) Requirement for CD44 in activated T cell extravasation into an inflammatory site. Science 278: 672–675. 9381175 10.1126/science.278.5338.672

[40] Gerdes J, Lemke H, Baisch H, Wacker HH, Schwab U, et al (1984) Cell cycle analysis of a cell proliferation-associated human nuclear antigen defined by the monoclonal antibody Ki-67. J Immunol 133: 1710–1715. 6206131

[41] Gerdes J, Schwab U, Lemke H, Stein H (1983) Production of a mouse monoclonal antibody reactive with a human nuclear antigen associated with cell proliferation. Int J Cancer 31: 13–20. 6339421 10.1002/ijc.2910310104

[42] Vincent BG, Young EF, Buntzman AS, Stevens R, Kepler TB, et al (2010) Toxin-coupled MHC class I tetramers can specifically ablate autoreactive CD8+ T cells and delay diabetes in nonobese diabetic mice. J Immunol 184: 4196–4204. 20220085 10.4049/jimmunol.0903931 PMC2868268

[43] Ciupe SM, Devlin BH, Markert ML, Kepler TB (2009) The dynamics of T-cell receptor repertoire diversity following thymus transplantation for DiGeorge anomaly. PLoS Comput Biol 5: e1000396. 19521511 10.1371/journal.pcbi.1000396 PMC2690399

[44] He M, Tomfohr JK, Devlin BH, Sarzotti M, Markert ML, et al (2005) SpA: web-accessible spectratype analysis: data management, statistical analysis and visualization. Bioinformatics 21: 3697–3699. 16051675 10.1093/bioinformatics/bti600

[45] Kepler TB, He M, Tomfohr JK, Devlin BH, Sarzotti M, et al (2005) Statistical analysis of antigen receptor spectratype data. Bioinformatics 21: 3394–3400. 15955781 10.1093/bioinformatics/bti539

[46] Yang Y, Charlton B, Shimada A, Dal Canto R, Fathman CG (1996) Monoclonal T cells identified in early NOD islet infiltrates. Immunity 4: 189–194. 8624809 10.1016/s1074-7613(00)80683-4

[47] Trudeau JD, Kelly-Smith C, Verchere CB, Elliott JF, Dutz JP, et al (2003) Prediction of spontaneous autoimmune diabetes in NOD mice by quantification of autoreactive T cells in peripheral blood. J Clin Invest 111: 217–223. 12531877 10.1172/JCI16409 PMC151866

[48] Sarukhan A, Gombert JM, Olivi M, Bach JF, Carnaud C, et al (1994) Anchored polymerase chain reaction based analysis of the V beta repertoire in the non-obese diabetic (NOD) mouse. Eur J Immunol 24: 1750–1756. 7519993 10.1002/eji.1830240805

[49] Sarukhan A, Bedossa P, Garchon HJ, Bach JF, Carnaud C (1995) Molecular analysis of TCR junctional variability in individual infiltrated islets of non-obese diabetic mice: evidence for the constitution of largely autonomous T cell foci within the same pancreas. Int Immunol 7: 139–146. 7718510 10.1093/intimm/7.1.139

[50] Liu CP (2006) Glutamic acid decarboxylase-specific CD4+ regulatory T cells. Ann N Y Acad Sci 1079: 161–170. 17130549 10.1196/annals.1375.025

[51] Komagata Y, Masuko K, Tashiro F, Kato T, Ikuta K, et al (1996) Clonal prevalence of T cells infiltrating into the pancreas of prediabetic non-obese diabetic mice. Int Immunol 8: 807–814. 8671670 10.1093/intimm/8.6.807

[52] Han B, Serra P, Yamanouchi J, Amrani A, Elliott JF, et al (2005) Developmental control of CD8 T cell-avidity maturation in autoimmune diabetes. J Clin Invest 115: 1879–1887. 15937548 10.1172/JCI24219 PMC1142112

[53] Galley KA, Danska JS (1995) Peri-islet infiltrates of young non-obese diabetic mice display restricted TCR beta-chain diversity. J Immunol 154: 2969–2982. 7533189

[54] Drexler K, Burtles S, Hurtenbach U (1993) Limited heterogeneity of T-cell receptor V beta gene expression in the early stage of insulitis in NOD mice. Immunol Lett 37: 187–196. 8258459 10.1016/0165-2478(93)90030-6

[55] Codina-Busqueta E, Scholz E, Munoz-Torres PM, Roura-Mir C, Costa M, et al (2011) TCR bias of in vivo expanded T cells in pancreatic islets and spleen at the onset in human type 1 diabetes. J Immunol 186: 3787–3797. 21325620 10.4049/jimmunol.1002423

[56] Baker FJ, Lee M, Chien YH, Davis MM (2002) Restricted islet-cell reactive T cell repertoire of early pancreatic islet infiltrates in NOD mice. Proc Natl Acad Sci U S A 99: 9374–9379. 12082183 10.1073/pnas.142284899 PMC123148

[57] Young EF, Hess PR, Arnold LW, Tisch R, Frelinger JA (2009) Islet lymphocyte subsets in male and female NOD mice are qualitatively similar but quantitatively distinct. Autoimmunity 42: 678–691. 19886740 10.3109/08916930903213993 PMC2874559

[58] Roep BO, Atkinson MA, van Endert PM, Gottlieb PA, Wilson SB, et al (1999) Autoreactive T cell responses in insulin-dependent (Type 1) diabetes mellitus. Report of the first international workshop for standardization of T cell assays. J Autoimmun 13: 267–282. 10479395 10.1006/jaut.1999.0312

[59] Kaufman DL, Tisch R, Sarvetnick N, Chatenoud L, Harrison LC, et al (2001) Report from the 1st International NOD Mouse T-Cell Workshop and the follow-up mini-workshop. Diabetes 50: 2459–2463. 11679422 10.2337/diabetes.50.11.2459

[60] Eisenbarth GS, Kotzin BL (2003) Enumerating autoreactive T cells in peripheral blood: a big step in diabetes prediction. J Clin Invest 111: 179–181. 12531872 10.1172/JCI17621 PMC151887

[61] Li L, He Q, Garland A, Yi Z, Aybar LT, et al (2009) beta cell-specific CD4+ T cell clonotypes in peripheral blood and the pancreatic islets are distinct. J Immunol 183: 7585–7591. 19917704 10.4049/jimmunol.0901587

[62] Lissina A, Ladell K, Skowera A, Clement M, Edwards E, et al (2009) Protein kinase inhibitors substantially improve the physical detection of T-cells with peptide-MHC tetramers. J Immunol Methods 340: 11–24. 18929568 10.1016/j.jim.2008.09.014 PMC3052435

[63] Liu Z, Cort L, Eberwine R, Herrmann T, Leif JH, et al (2012) Prevention of type 1 diabetes in the rat with allele-specific anti-T cell receptor antibody. Diabetes 61: 1160–1168. 22368175 10.2337/db11-0867 PMC3331757

