# Supplementary figures and images for: Autoreactive Effector/Memory CD4+ and CD8+ T Cells Infiltrating Grafted and Endogenous Islets in Diabetic NOD Mice Exhibit Similar T Cell Receptor Usage
Source: PLoS One. 2012 Dec 14;7(12):e52054. doi: 10.1371/journal.pone.0052054 (PMC3522632; doi:10.1371/journal.pone.0052054)

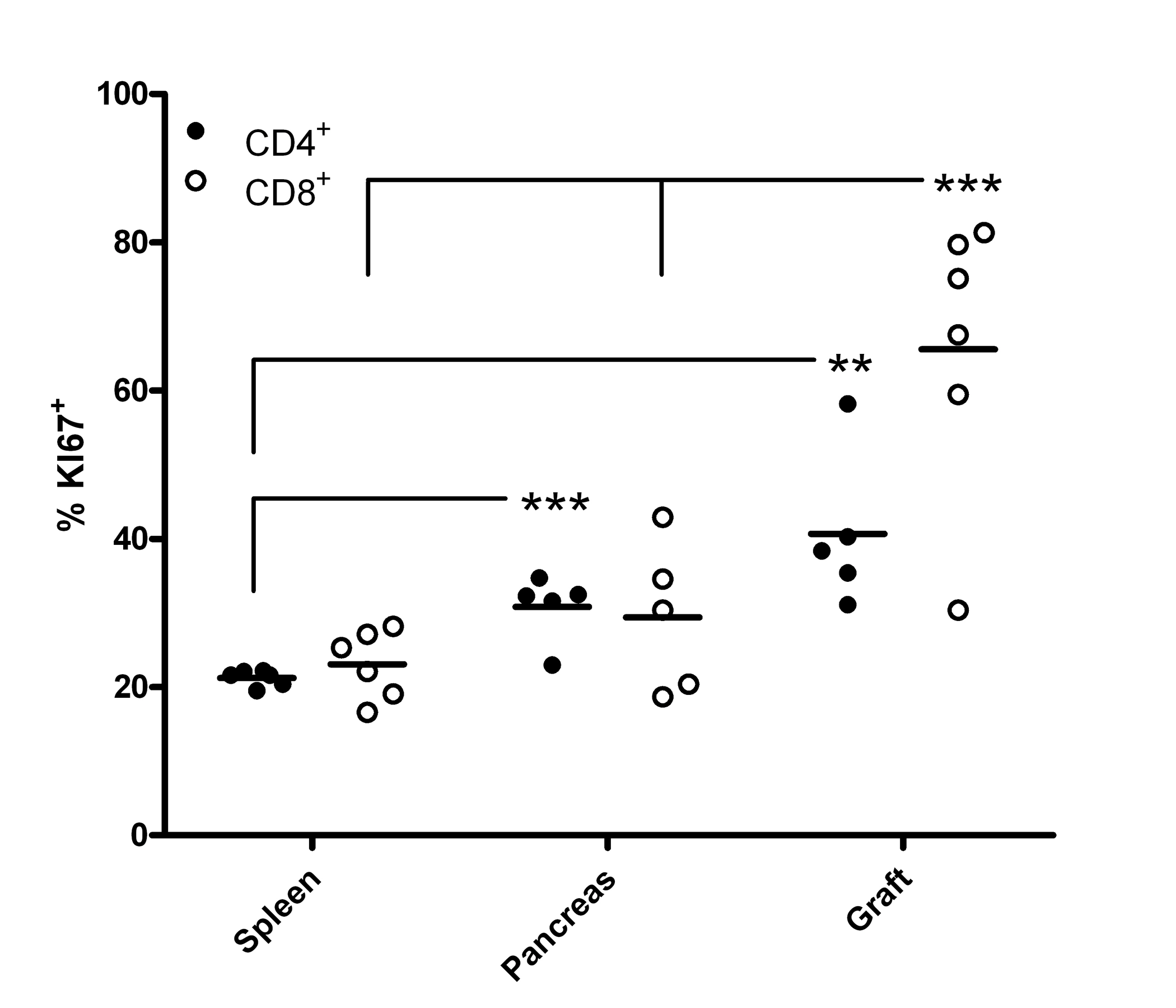

Supplement: Figure S1 — Increased proliferation of islet graft-infiltrating Teff/mem in individual NOD recipients. The frequency of Ki67-staining CD4+ and CD8+ Teff/mem was determined in the spleen, islet graft and pancreas of individual NOD recipients. ***p<0.001, **p<0.01; Student’s t test. (TIFF) [file pone.0052054.s001.tiff]
